# Comment on “Heterologous adenovirus‐vector/messenger RNA regimen, humoral response and liver transplant recipients”

**DOI:** 10.1002/hep4.2071

**Published:** 2022-08-23

**Authors:** Pathum Sookaromdee, Viroj Wiwanitkit

**Affiliations:** ^1^ Private Academic Consultant Bangkok Thailand; ^2^ Honorary Professor, Dr. D. Y. Patil University Pune India

The article “Heterologous adenovirus‐vector/messenger RNA regimen is associated with improved severe acute respiratory syndrome coronavirus 2 humoral response in liver transplant recipients” provides important data to support coronavirus disease 2019 (COVID‐19) vaccination for liver transplant recipients. According to Mendizabal et al.,^[^ ^1^ ^]^ rAd26/mRNA‐1273 was independently associated with a higher antibody response. We agree that heterologous vaccination might increase immunogenicity. However, alternative considerations are warranted. Prior infection, not accounted for in the analysis, could impact subsequent immunity. In most cases, asymptomatic COVID‐19 infection is not unusual.^[^ ^2^ ^]^ A history of infection cannot exclude COVID‐19 in the absence of any symptoms. Prior infection could raise antibody levels^[^ ^3^ ^]^ and be misinterpreted as the effect of vaccination.

## CONFLICT OF INTEREST

Nothing to report.
